# *KRAS* oncogene may be another target conquered in non‐small cell lung cancer (NSCLC)

**DOI:** 10.1111/1759-7714.13538

**Published:** 2020-10-06

**Authors:** Hanxiao Chen, Jun Zhao

**Affiliations:** ^1^ Key Laboratory of Carcinogenesis and Translational Research (Ministry of Education/Beijing), Departments of Thoracic Medical Oncology Peking University Cancer Hospital and Institute Beijing China

**Keywords:** *KRAS* mutation, non‐small cell lung cancer, target, therapy

## Abstract

Kirsten rat sarcoma viral oncogene homolog (*KRAS*) is one of the most common mutant oncogenes in non‐small cell lung cancer (NSCLC). The survival of patients with *KRAS* mutations may be much lower than patients without *KRAS* mutations. However, due to the complex structure and diverse biological properties, it is difficult to achieve specific inhibitors for the direct elimination of KRAS activity, making KRAS a challenging therapeutic target. At present, with the tireless efforts of medical research, including KRAS G12C inhibitors, immunotherapy and other combination strategies, this dilemma is expected to an end. In addition, inhibition of the downstream signaling pathways of KRAS may be a promising combination strategy. Given the rapid development of treatments, understanding the details will be important to determine the individualized treatment options, including combination therapy and potential resistance mechanisms.

## Abstract 2

The survival of patients with KRAS mutations may be much lower than patients without KRAS mutations. At present, with the tireless efforts of medical research, including KRAS G12C inhibitors, immunotherapy and other combination strategy, this dilemma of KRAS mutated NSCLC is expected to an end.

## Introduction

Lung cancer, known as the most common cancer worldwide, remains a leading cause of cancer related deaths around the world, including China.^1^ Non‐small cell lung cancer (NSCLC) accounts for 80% with the majority of patients diagnosed at an advanced stage, without the opportunity for radical resection or radiotherapy.^2^ For these patients, traditional chemotherapy, even combined with a third antiangiogenic drug (eg, bevacizumab) and maintenance therapy, could slightly prolong overall survival (OS) to 20 months, relieving symptoms and simultaneously improving their quality of life.^3^, ^4^, ^5^

During the previous decades, numerous genetic variations have been described in NSCLC, including epidermal growth factor receptor (EGFR), *KRAS* and anaplastic lymphoma kinase (*ALK*), as the most commonly altered oncogenes acting as tumor driver genes.^6^ Fortunately, target therapies in patients with *EGFR* sensitive mutation (eg, gefetinib, osimertinib), *ALK* or ROS proto‐oncogene 1 (ROS1) gene fusion have significantly improved survival time, with median OS of three years or more.^7^, ^8^ In contrast, NSCLC patients with *KRAS* mutation do not respond to the EGFR tyrosine kinase inhibitors (TKIs) mentioned above, even those with concurrent sensitive EGFR mutation, which could be attributed to continuous activation of the downstream Raf‐MEK‐ERK pathway.^9^ Even worse, due to the particularity of the KRAS protein structure, almost no drugs are able to directly target KRAS, causing KRAS to be an unconquerable fortress in previous decades. In addition, NSCLC patients with *KRAS* mutation respond poorly to traditional chemotherapy, leading to worse prognosis compared to the wild‐type group.^10^, ^11^ However, after years of research, strategy against KRAS has gone “from worse to bad, to better”. Recently, breakthroughs have been made in the development of KRAS‐targeted drugs, including AMG‐510, MRTX849 and other treatment such as immunotherapy, although the optimal treatment for *KRAS*‐mutated NSCLC patients has not yet been discovered. There is therefore an urgent clinical need to review the prognostic and predictive role of KRAS in NSCLC patients. In this study, we focus on the molecular biology, clinicopathological features and treatment progress of *KRAS* gene mutation, in order to improve our understanding of *KRAS*‐mutant NSCLC.

## Molecular and clinicopathological features

KRAS, first described in NSCLC in 1984 by Santos *et al*. are intracellular guanine nucleotide binding proteins (G proteins), belonging to the family of GTPases.^12^ The KRAS proteins perform as RAS‐guanosine triphosphate (GTP) (active form) and RAS‐guanosine diphosphate (GDP) (inactive form) status. When the extracellular growth factors (eg, epidermal growth factor [EGF]) transmit the signal to downstream KRAS protein, the binding activity to GTP is enhanced, making the KRAS protein bind to GTP as an active form (RAS‐GTP complex). The signaling system such as Raf‐MEK‐ERK, the phosphoinositol 3 kinase (PI3K)‐protein kinase B (AKT)‐mammalian target of rapamycin (mTOR) and RalGDS‐RalA/B pathways or the TIAM1‐RAC1 pathway are open, stimulating tumor cells to grow, proliferate and spread, not affected by upstream signals from EGFR.^13^, ^14^, ^15^

Nine subtypes of *KRAS* mutation have been identified in the Chinese population. Generally, *KRAS* mutations which affect exons 2 and 3 are the most common, with G to C transition in codons 12 or 13, resulting in G12C (GGT → TGT) mutations (33.6%), followed by G12D (GGT → GAT) (23.9%), G12V (GGT → GTT) (22.1%), and G12A (GGT → GCT) mutation (7.1%).^16^ The mutation rate of *KRAS* in lung adenocarcinoma ranges from 15% to 30%, including 12% of G12C.^10^, ^17^ A higher frequency has been identified in western populations (20%–25% vs. 10%–15%).^11^ In NSCLC patients, *KRAS* mutations are more common in young women diagnosed with adenocarcinoma who have a history of smoking.^18^ However, in another study involving 1368 Chinese patients, *KRAS* mutations have been found to be more common in males.^19^ Smoking history is generally believed to be a related factor. Incidence of *KRAS* mutations has been reported to reach between 25%–35% in smokers and 5% in nonsmokers.^20^ In addition, never‐smokers are more likely to have G12D mutation (56%), and G12C is more common among former and current smokers (41%).^21^ The different gene mutations also reflect biological heterogeneity, suggesting KRAS can activate different signal pathways. G12V and G12C mutations are associated with enhanced downstream RalA/B signaling pathway, and G12D mutations are more likely to activate the PI3K and MEK pathway.^22^ In terms of metastatic sites, *KRAS*‐mutated patients are more likely to present with brain and lung metastases. Pleuro‐pericardial metastases are more common in patients with G12V mutations, while bone metastases occur more often in patients harboring the G12C mutation.^23^, ^24^

Generally, *KRAS* mutations are speculated to be mutually exclusive with *EGFR* mutations and EML4‐ALK translocations. Recently, cases harboring *KRAS* mutations coexisting with EGFR or ALK have been reported more frequently, suggesting *KRAS*‐mutant NSCLC may be a molecularly diverse entity.^25^ Ulivi *et al*. detected *EGFR*, *KRAS* and *ALK* genes in 282 patients simultaneously, and found that coexisting *EGFR*/*KRAS* or *ALK*/*KRAS* mutations accounted for 1.1% and 2.5%, respectively.^26^ Apart from those driver genes mentioned above, other genes such as TP53 (42%), STK11 (29%) and KEAP1/NFE2L2 (27%), also significantly correlate with *KRAS* mutations (co‐mutation), as suggested in a clinical study which included 330 patients with advanced *KRAS*‐mutant lung cancers.^27^

## *KRAS* mutation as a prognostic factor

Whether KRAS could be defined as a prognostic factor of NSCLC remains controversial, due to heterogeneity among different studies. Multiple meta‐analyses have been conducted in view of the disparity from individual studies. A meta‐analysis from 28 studies including 3620 patients suggested a lower survival rate of *KRAS‐*mutated adenocarcinoma (HR = 1.35, 95% CI: 1.16–1.56, *P* = 0.01), while not in squamous cell carcinoma.^28^ Coincidentally, another meta‐analysis from 41 studies including 13 103 patients (2374 *KRAS* positive) showed a worse OS when *KRAS* mutations were present (HR 1.56, 95% CI: 1.39–1.76, *P* =  0.00).^29^ While in a pooled analysis, data collected from several clinical trials (ANITA, IALT, JBR.10, and CALGB‐9633) showed a totally different outcome, suggesting no significant differences in prognostic value, even in subgroups divided by histology (HR 1.17, 95% CI: 0.96 to 1.42, *P* = 0.12).^18^ Therefore, the prognostic role of KRAS is a question which remains to be answered. Furthermore, different subtypes of *KRAS* mutation also determine its prognostic utility. In a study involving 677 patients, Yu *et al*. suggested that patients with codon 13 mutation had an increased risk of death (HR = 1.50, 95% CI: 1.11–2.04, *P* = 0.009), while there was no statistically significant difference between the patients with G12C/G12V mutation and other mutation subtype (*P* =  0.74).^30^

On the other hand, as mentioned above, *KRAS* mutation has been found to co‐occur with TP53, STK11/LKB1 or CDKN2A in a large proportion of patients. When co‐mutated with STK11/LKB1, not TP53, patients have been reported to suffer even worse survival, as previously reported in a clinical study.^31^

## *KRAS* mutation as a predictive factor

### Predictive significance of *KRAS* mutation on the efficacy of EGFR‐TKIs

Being a downstream gene of EGFR, *KRAS* mutation could cause the downstream Raf‐ERK‐MEK pathway to persistently activate, leading to reduced efficacy of EGFR‐TKIs. Two published meta‐analyses have shown lower response rates of EGFR TKIs, but no impact on survival in patients with *KRAS*‐mutant NSCLC.^32^, ^33^ Several prospective studies have shown that *KRAS* mutations predict poor survival and efficacy of EGFR‐TKIs.^34^ In addition, particular *KRAS* mutation subtypes may lead to different prognosis. Zer *et al*. conducted a pooled analysis of 275 patients with *KRAS* mutation treated with EGFR‐TKIs, which showed that patients with G12C/G12V mutation had a poor prognosis, while G12D/G12S positive patients could benefit from EGFR‐TKIs.^35^ Similarly, Fiala *et al*. showed that patients harboring G12C KRAS mutation had shorter progression‐free survival (PFS) and OS than those with non‐G12C *KRAS* mutations who were treated with EGFR‐TKIs, suggesting that non‐G12C *KRAS* mutations may act as wild‐type *KRAS* and wild‐type *EGFR* genotype.^36^

### Predictive significance of *KRAS* mutation on the efficacy of chemotherapy

Cytotoxic chemotherapy is still recommended as the standard therapy for NSCLC patients with *KRAS* mutation. As far back as 1990, *KRAS* mutation was described as a negative prognostic marker for both OS and disease‐free survival (DFS) in lung cancer.^37^ Whether *KRAS* mutation could be evaluated as a predictive factor to select patients for chemotherapy regimens remains highly controversial. Some researchers tend to believe that there is no relationship between *KRAS* mutation and therapeutic response. An IFCT‐0002 trial included stage I and II NSCLC patients to compare TC regimen (carboplatin and paclitaxel) and GC regimen (cisplatin and gemcitabine) in pre‐ or perioperative chemotherapy. Univariate analyses showed that KRAS status was associated with ORR. However, this association was not significant in the multivariate analysis. In the TRIBUTE trial, which compared first‐line paclitaxel/carboplatin plus erlotinib or placebo in advanced NSCLC patients, the objective response rate (ORR), time to progression (TTP) and OS did not differ according to *KRAS* mutation status.^38^ Even recently, in a retrospective clinical study which included 161 patients treated with platinum‐based chemotherapy in first‐line setting, *KRAS* mutation was not predictive for worse response to chemotherapy, neither for PFS nor OS.^39^ Nonetheless, other researchers such as Metro *et al*. suggest that *KRAS* mutation appears to negatively affect sensitivity to first‐line platinum‐based chemotherapy in patients with advanced nonsquamous and *EGFR* wild‐type NSCLC, including ORR, disease control rate (DCR) and survival. In terms of subtypes, patients with mutations at codon 13 may perform worse than codon 12 even without statistical significance.^40^ In a study comparing clinical outcome after first‐line platinum‐based chemotherapy in *KRAS*‐mutated NSCLC, significantly improved ORR (*P* < 0.01) was observed for taxanes in patients with G12V, but not PFS or OS.^41^ In the Chinese population, Jia *et al*. found that *KRAS*‐mutant NSCLC had a significantly shorter PFS. It has also been reported that patients with *KRAS* G12V mutation had the poorest PFS compared with non‐G12V mutant cases (*P* = 0.045).^24^

Overall, there is still no evidence confirming the predictive value of *KRAS* mutations in stage IV or earlier stage for specific chemotherapy regimens.

### Predictive significance of *KRAS* mutation on the efficacy of immunotherapy

Studies have reported that smoking‐related lung cancers are significantly associated with greater tumor mutation burden (TMB) and programmed death ligand 1 (PD‐L1) expression. Therefore, because of the correlation with smoking history, we suggest that *KRAS*‐mutant NSCLC may express a higher level of PD‐L1 protein and TMB compared with wild‐type tumors, which may reflect the efficacy of immune checkpoint inhibitors (ICIs) against PD‐1/PD‐L1. Confirming our conjecture, Scheel *et al*. found that PD‐L1 expression was highest in the tumor specimens with mutant *KRAS*, mutant TP53 and wild‐type STK11.^42^ Corresponding with previous studies, in a recent KEYNOTE‐189 trial, PD‐L1 expression and TMB level of tumor tissue (tTMB) tended to be higher among patients with *KRAS* mutations.^43^

Chen *et al*. investigated the functional significance of PD‐1/PD‐L1 blockade in *KRAS*‐mutant lung adenocarcinoma. They found that PD‐L1 was upregulated by *KRAS*‐G12D mutation through pERK signaling, inducing the apoptosis of CD3+ T cells. Blockade of PD‐1/PD‐L1 pathway may be a promising therapeutic strategy for *KRAS*‐mutant lung adenocarcinoma.^44^ Recently, research conducted by Liu *et al*. indicated that *KRAS* mutations are associated with an inflammatory tumor microenvironment and tumor immunogenicity, together with an increased proportion of PDL1/CD8 tumor infiltrating lymphocytes (TILs), which may reflect a better response to ICIs.^45^

To understand the relationship between ICIs and oncogenic driver genes, a retrospective study was conducted from the IMMUNOTARGET registry. In certain subgroups, driver genes were found to be positively associated with PD‐L1 expression (KRAS, EGFR) and PFS was longer (KRAS, cMET).^46^ In another retrospective study including 282 patients treated with immunotherapy, PD‐L1 expression seemed to be more relevant for predicting the efficacy of ICIs in *KRAS*‐mutant NSCLC than in wild‐type NSCLC. However, there was no significant difference reported in ORR, PFS and OS in terms of *KRAS* mutation status, or in the mutation subtypes.^47^ In 2016, Dong *et al*. reported that TP53 and *KRAS* mutations could be potential predictors of anti‐PD‐1/PD‐L1 therapy.^48^ Cinausero *et al*. also found that *KRAS*‐mutant patients had a better response to PD‐1 inhibitors than patients with *KRAS* wild‐type.^49^ A meta‐analysis in 2017 involving 3025 patients showed that immunosuppressants prolonged the OS of the *KRAS*‐mutant subgroup (HR = 0.65, *P* = 0.03).^50^ In phase III trials, PD‐1 antibody (nivolumab) or PD‐L1 antibody (atezolizumab) could improve survival to varying degrees in *KRAS*‐mutant chemorefractory NSCLC patients.^51^, ^52^ Recently, new findings on *KRAS* positive patients from the KEYNOTE‐042 trial were presented at the European Society for Medical Oncology (ESMO) Immuno‐Oncology Congress. Clinical data from this exploratory analysis showed that pembrolizumab reduced the risk of death by 58% (HR = 0.42, 95% CI: 0.22–0.81) in patients with any *KRAS* mutation and by 72% (HR = 0.28, 95% CI: 0.09–0.86) in patients with *KRAS* G12C mutation compared to chemotherapy. In addition, in the pembrolizumab arm, ORR and PFS was significantly elevated in the *KRAS*‐mutant population than the wild‐type population (56.7% vs. 29.1% and 12 vs. 6 months, respectively).^53^

Other clinical trials for *KRAS*‐mutant lung adenocarcinoma patients exploring the benefits of ICIs are still ongoing (NCT03777124, etc). It is anticipated that ICIs will bring new hope for *KRAS‐*mutant NSCLC patients in the future.

## Target therapy for *KRAS*‐mutant NSCLC

### Target therapy inhibiting *KRAS* downstream pathway of KRAS

Due to the higher incidence of *KRAS* mutation in NSCLC, more and more attention is being paid to the therapy. However, the development of new drugs which directly inhibit *KRAS* has been challenging because of the complexity of *KRAS* biochemistry, such as SML‐8‐73‐1 (compounds that target the guanine nucleotide binding pocket) or ARS‐853 (allele‐specific inhibitors).^54^ As mentioned above, *KRAS* mutation could cause constitutive activation of KRAS, leading to the persistent stimulation of downstream signaling pathways that promote tumorigenesis. Therefore, inhibition of the downstream pathways of KRAS may be alternatives, including Raf‐MEK‐ERK and PI3K‐AKT‐mTOR pathways.

### BRAF inhibitors

BRAF plays an important role in regulating the MAPK/ERK signaling pathway, affecting cell division, differentiation and secretion, with a mutation rate of 1%–4% in NSCLC.^55^, ^56^ BRAF inhibitors such as vemurafenib and dabrafenib have been approved to treat *BRAF*‐mutant melanoma, and are recommended for *BRAF*‐mutant NSCLC.^57^, ^58^, ^59^ However, there is still no data to demonstrate their clinical efficacy in *KRAS*‐mutant NSCLC, since *KRAS* and *BRAF* mutation are usually mutually exclusive. Other Raf inhibitors may be alternatives. Sorafenib is a multikinase inhibitor with a dual antitumor effect enabling it to block the Raf/MEK/ERK pathway and inhibit VEGFR and PDGFR.^60^ In the BATTLE study, a prospective phase II trial, sorafenib achieved a DCR of 61%.^61^ In another phase II study for chemorefractory NSCLC patients with *KRAS* mutation, six week DCR was 52.6% and median PFS was 2.3 months.^62^ In the phase III MISSION trial, sorafenib prolonged PFS in the *KRAS*‐mutant subgroup compared with the placebo in the pretreated nonsquamous NSCLC patients, with OS failed.^63^ There is still no sufficient evidence for the use of Raf inhibitors.

### MEK inhibitors

Working as a downstream effector of mitogen‐activated protein kinase (MAPK) pathway, MEK may be a suitable target. Preclinical data has suggested that inhibition of MEK1/2 could be an effective strategy for the treatment of tumors driven by *KRAS* mutations.^64^, ^65^, ^66^ However, the efficacy of MEK inhibitors as monotherapy such as CI‐1040,^67^ RO5126766^68^ and PD‐0325901^69^ in clinical trials is limited, due to activation of compensatory signaling effectors.

Selumetinib (AZD6244) is a potent and highly selective MEK1/2 inhibitor, showing antitumor activity in xenograft tumors in preclinical studies.^70^ Similarly, single agent therapy did not show superiority over chemotherapy in several studies, suggesting a need to explore combination approaches.^71^ In a phase II study, the combination regimen of selumetinib and docetaxel showed an increased median PFS of 5.3 and ORR of 37%,^72^ whereas in a follow‐up randomized phase III (SELECT‐1) study, combining selumetinib with docetaxel did not improve the OS and PFS of *KRAS*‐mutant NSCLC patients, compared with docetaxel.^73^ In a randomized phase II trial in patients with *KRAS* positive and negative NSCLC, 11 patients received selumetinib alone and 30 patients received erlotinib and selumetinib. Unfortunately, in the *KRAS*‐mutant cohort, the combination did not improve ORR, PFS, and OS. Instead, more serious adverse events (AEs) were found in patients treated with combination therapy. Interestingly, there was an increased level of expression of PD‐1 on CD8+ cells after treatment with selumetinib, suggesting benefits from the combination with PD‐1/L1 antibody.^74^ Although there is no evidence to prove the survival advantages, some clinical trials are still underway to investigate the combination approaches (NCT03004105, NCT03299088).

Trametinib was initially approved for the treatment of metastatic melanoma with BRAF V600E or V600K mutations, belonging to the same molecular class as selumetinib. As monotherapy, trametinib has shown limited efficacy and similar survival outcome compared with docetaxel.^75^ In several phase I studies, combination of trametinib and docetaxel/pemetrexed showed a DCR of approximate 60%.^76^, ^77^ Data from a phase II study presented in ASCO 2019 showed an ORR of 33% in patients with *KRAS* mutation. The median PFS was 4.1 months and median OS was 11.1 months. Subgroup analysis showed that the efficacy of patients with non‐G12C mutations was better, including ORR (37% vs. 26%), PFS (4.1 vs. 3.3 months) and OS (16.3 vs. 8.8 months) (Abstract #9021). In conjunction with therapeutic blockade of the PI3K/mTOR pathway may be another available strategy due to extensive crosstalk between both pathways. However, clinical outcomes showed minimal activity in *KRAS*‐mutant NSCLC receiving pan‐PI3K inhibitor buparlisib (BKM120) and trametinib.^78^

Other MEK inhibitors, such as binimetinib (MEK162), PD‐0325901 and RO4987655, have shown their safety in phase I trials.^79^, ^80^, ^81^ However, clinical trials assessing the efficacy against *KRAS*‐mutant NSCLC are still underway (NCT02276027, NCT02022982, NCT02039336). In general, emphasis of MEK inhibitors would be combination therapy, especially with chemotherapy, which should be validated in phase III clinical studies.

### mTOR inhibitors

The PI3K/Akt/mTOR pathway is a parallel signal transduction pathway, which was thought to be a target for *KRAS*‐mutant NSCLC due to preclinical data.^82^ However, single mTOR inhibitor such as ridaforalimus failed to prove its efficacy even with a trend of better OS and PFS in the ridaforolimus arm.^83^ Dual PI3K–mTOR inhibitors such as NVP‐BEZ235 also failed to demonstrate their preclinical efficacy. Dual‐targeting strategy involving PI3K/ AKT/mTOR and Ras/MEK/ERK pathways was another option on account of the synergy between the two. Phase I clinical trials have been conducted proving the endurance with early signs of anticancer activity in unselected solid tumors.^84^, ^85^ However, no preliminary data in the *KRAS*‐mutant population have been reported.

### Focal adhesion kinase (FAK) inhibitors

FAK is a downstream effector of KRAS signaling, playing an important role in cell migration. Mutation of *KRAS* could cause the activation of tumor suppressor genes INK4a/ARF/p16, leading to hyperactivation of the GTPase RHOA by MEK1/2 and ERK1/2.^86^ Inhibition of the RHOA‐FAK pathway could be a potential strategy. Preclinical data show that FAK inhibition leads to sustained DNA damage in mutant *KRAS* NSCLC cells.^87^ Defactinib (*VS*‐6063) is a second‐generation inhibitor of FAK. In a phase 2 clinical trial, *KRAS*‐mutant NSCLC patients were assigned to defactinib 400 mg orally b.i.d. based on TP53 and CDKN2A mutation status, showing the PFS rate at 12 weeks of 31% after extensive pretreatment with well tolerated side effects.^88^ Currently, a clinical trial focusing on combination therapy with CH5126766 is being planned the results of which are to be expected in the future.

### Heat shock proteins (HSP) 90 inhibitors

As a molecular chaperone, HSP90 assists in the folding and maturation of different types of oncoproteins, which play an important role in tumor formation and growth. Inhibition of HSP90 was thought to be another potent therapeutic target by disrupting proper functioning of oncogenic proteins, including EGFR, HER‐2 and EML4‐ALK.^89^ The preclinical trial of the HSP90 inhibitor, ganetespib, has shown the activity of cell apoptosis in *KRAS*‐mutant cell lines.^90^ In a phase II clinical study, ganetespib was recently studied as monotherapy in previously treated NSCLC patients, and among 17 patients with KRAS mutations, 47% were reported to have tumor shrinkage.^91^ However, combination studies with chemotherapy failed to improve OS or PFS for *KRAS*‐mutant NSCLC in phase II clinical trials.^92^ In addition, in a recently published phase III trial, adding ganetespib to docetaxel also failed to improve survival in advanced lung adenocarcinoma.^93^ Other combination strategies with PI3K/mTOR or MEK inhibitors may be desirable.

### *KRAS* inhibitors

As previously described, G12C mutations account for almost 50% of the *KRAS*‐mutant NSCLC population. Therefore, drugs targeting the G12C variant could have a major therapeutic impact. The mutant cysteine of *KRAS* G12C is adjacent to a pocket (P2) of the inactivated KRAS. Thus, many covalent inhibitors have been focused on the development.^94^

ARS‐853 is a selective, covalent inhibitor of KRAS G12C, being the first direct KRAS inhibitor. In cell models, ARS‐853 and its analogues covalently interact with the GDP‐bound mutant KRAS G12C protein, transforming it into an “inactivated” conformation. However, pharmacokinetics (PK) results have not been verified in vivo due to an adequate drug threshold needed in KRAS cycles.^54^ Based on the ARS‐853, ARS‐1620 was synthesized and proved to be 10 times more active than the former. Further analysis has indicated excellent bioavailability of ARS‐1620 in a mouse model. In addition, in an NSCLC patient‐derived xenograft with *KRAS* G12C mutation, ARS‐1620 showed tumor regression, and effectively inhibited the downstream ERK phosphorylation and activated apoptosis.^95^

The surface of KRAS‐G12C has a groove formed by the steering of His95, which could be occupied by aromatic hydrocarbons to promote binding to KRAS G12C protein. Among numerous screening drugs, AMG 510, which is structurally related and overlapped with ARS‐1620, is the preferred candidate for binding to His95 grooves.^96^ in vitro and in vivo, AMG 510 has been reported to show remarkable antitumor effects.^97^ Being the first G12C inhibitor in the clinic, 76 patients with *KRAS* G12C mutations, most of whom had previously received at least two‐lines of therapy, were enrolled in an open‐label phase I study of AMG 510 (NCT 03600883). Among the 23 NSCLC patients with evaluable efficacy in all dose cohorts, 11 had tumor shrinkage (PR), reaching the ORR of 48% and DCR of 96%, and among the 13 evaluable patients treated with 960 mg AMG 510, seven had tumor shrinkage (PR), reaching the ORR of 54% and DCR of 100%.^98^ In addition, dose‐limited toxicity (DLT) has to date not been reported. To further achieve a better effect, a series of attempts have been made with dual‐drug combination. AGM 510 and a SHP2 inhibitor, RMC‐4630, have been combined to treat advanced solid tumor with *KRAS* G12C mutation. In vitro, AMG 510 combined with RMC‐4550, another SHP2 inhibitor, showed a strong synergistic effect on tumor cells.^97^ Early efforts have also been made with regard to the combination of AMG 510 and ICIs. A preclinical study based on the combination of AMG 510 and pembrolizumab showed that tumors disappeared permanently in 9/10 mice, suggesting an acquired immune response to AMG 510/pembrolizumab therapy. The phase 1/2 study combining AMG 510 with PD1/PDL1 inhibitor (NCT# 04185883) is currently ongoing. Furthermore, other combination drugs such as MAPK signaling pathway inhibitors are under investigation.

MRTX849 is a specifically optimized oral inhibitor, keeping KRAS G12C in its inactive GDP‐bound status and inhibiting KRAS‐dependent signaling pathways in *KRAS* G12C mutant solid tumor. In vivo, MRTX 849 has displayed broad‐spectrum antitumor activity in *KRAS*‐mutant solid tumors, including NSCLC.^99^ With regard to the phase I/II clinical trial named MRTX849‐001 currently ongoing (NCT 03785249), MRTX849 safety and antitumor activity has been reported in patients with NSCLC, colorectal cancer (CRC) and appendiceal cancers with *KRAS* G12C mutation. Preliminary clinical data was presented at the 2019 AACR‐NCI‐EORTC meeting, which showed ORR of 60% (three of five evaluable NSCLC) at the highest dose of 600 mg b.i.d., with one DLT.^100^ Similar to AMG 510, combination therapy has been conducted in vitro using MRTX 849 and other target drugs in order to enhance the efficacy of MRTX 849 and overcome potential resistance. The combined small molecular inhibitors included afatinib targeting the HER family, CD4/6 inhibitor palbociclib and SHP2 inhibitor RMC4450.^99^

Studies on other new drugs targeting KRAS are still ongoing, including BI 1701963, a pan‐KRAS inhibitor and LY3499446 (NCT #04165031) in phase I clinical trials (Table 1). However, there are still problems to be taken into consideration. For example, current clinical trials have excluded patients with active or even stable CNS metastasis, lacking clinical data of KRAS inhibitor penetration. In addition, there are still patients with other *KRAS* mutations such as G12D/G12V or concurrent mutation of other genes such as TP53, for whom further clinical decision‐making should be considered.

**Table 1:** Novel inhibitors targeting *KRAS* and the clinical trials

Agents	NCT clinical trial	Phase	Subjects	Status
AMG 510 (+/− anti PD‐1/L1)	03600883	1/2	Monotherapyin subjects with advanced solid tumors with *KRAS* G12C mutation; combination therapy in subjects with advanced NSCLC with G12C mutation (anti PD‐1/L1)	Recruiting
MRTX 849 (+/− pembrolizumab/ cetuximab/afatinib)	03785249	1/2	Monotherapy or combination therapy in advanced solid tumors that have a *KRAS* G12C mutation	Recruiting
LY3499446 (+/− abemaciclib/ cetuximab/erlotinib/ docetaxel	04165031	1/2	Advanced solid tumors with *KRAS* G12C mutation	Suspended
JNJ‐74699157	04006301	1	Solid tumor with *KRAS* G12C mutation. The subject received or was ineligible for standard treatment options	Active, not recruiting
BI 1701963/+ trametinib	04111458	1	Solid tumors with *KRAS* mutation	Recruiting

In conclusion, although KRAS has previously been defined as an untreatable target, especially G12C mutation, clinical data of AMG 510/MRTX 849 targeting KRAS and new combined strategies targeting the downstream signaling pathways of KRAS or related pathways appear promising. In addition, immune checkpoint inhibitors have been proven effective in *KRAS*‐mutated NSCLC. Faced with numerous choices, future investigation should focus on selecting appropriate drugs for appropriate population and resistance mechanisms of first generation KRAS G12C inhibitors.
